# Assessment of quality of routine health information system data and associated factors among departments in public health facilities of Harari region, Ethiopia

**DOI:** 10.1186/s12911-021-01651-2

**Published:** 2021-10-19

**Authors:** Adisu Tafari Shama, Hirbo Shore Roba, Admas Abera Abaerei, Teferi Gebru Gebremeskel, Negga Baraki

**Affiliations:** 1 Department of Public Health, Institute of Health Sciences, Wollega University, P. O. Box: 395, Nekemte, Ethiopia; 2 School of Public Health, College of Health and Medical Sciences, Haramaya University, Harar, Ethiopia; 3 Department of Reproductive Health, College of Health Sciences, Aksum University, Aksum, Ethiopia

**Keywords:** Data quality, Routine health information system, Health facilities, Departments, Harari region, Ethiopia

## Abstract

**Background:**

Despite the improvements in the knowledge and understanding of the role of health information in the global health system, the quality of data generated by a routine health information system is still very poor in low and middle-income countries. There is a paucity of studies as to what determines data quality in health facilities in the study area. Therefore, this study was aimed to assess the quality of routine health information system data and associated factors in public health facilities of Harari region, Ethiopia.

**Methods:**

A cross-sectional study was conducted in all public health facilities in the Harari region of Ethiopia. The department-level data were collected from respective department heads through document reviews, interviews, and observation checklists. Descriptive statistics were used to data quality and multivariate logistic regression was run to identify factors influencing data quality. The level of significance was declared at *P* value < 0.05.

**Result:**

The study found good quality data in 51.35% (95% CI 44.6–58.1) of the departments in public health facilities in the Harari Region. Departments found in the health centers were 2.5 times more likely to have good quality data as compared to those found in the health posts. The presence of trained staffs able to fill reporting formats (AOR = 2.474; 95% CI 1.124–5.445) and provisions of feedbacks (AOR = 3.083; 95% CI 1.549–6.135) were also significantly associated with data quality.

**Conclusion:**

The level of good data quality in the public health facilities was less than the expected national level. Lack of trained personnel able to fill the reporting format and feedback were the factors that are found to be affecting data quality. Therefore, training should be provided to increase the knowledge and skills of the health workers. Regular supportive supervision and feedback should also be maintained.

**Supplementary Information:**

The online version contains supplementary material available at 10.1186/s12911-021-01651-2.

## Background

The health information system (HIS) is one of the six building blocks of a health system designed for the generation and use of information for other functions of the health system [1]. The purpose of a health information system is to routinely generate quality health data that provides specific evidence support to make decisions on health issues [2]. In the “One plan, one budget, and one report” policy of Ethiopia, HIS is the core information system [3]. The information revolution is one of the four big agendas of Ethiopia’s Health sector transformation plan II (HSTP-II) and it is the phenomenal advancement in the methods and practice of collecting, analyzing, presenting, and disseminating information. Data quality, defined as data's fitness to serve its purpose in a given context in terms of accuracy, completeness, and timeliness [4],-is an essential element of this information revolution agenda [5].

Routine health care data have no importance unless it is accurate, processed, and used to inform decisions hence responsive to the local situations [6]. Improved health system performance is directly linked with the quality and use of routine data in a country’s HIS [5, 7].

Despite the improvements in the knowledge and understanding of the role of health information in the global health system, the quality of data generated by routine HIS is still very poor in low and middle-income countries [8]. The quality of data was found to be between 34 and 72% in many African countries [9]. The large volume and variety of data generated in public health facilities are overlooked due to their limited qualities [10–13].

In Ethiopia, routine health information systems data quality problem is for most indicators [14], and data quality is below the 80% national expectation [15]. The data completeness, accuracy, and timeliness were found to be between 33 and 78% in different parts of the country [4, 5, 16–19]. In Addis Ababa, the overall data quality was found to be between 57.9 and 76.22% [20, 21] whereas it was 75.3% in Dire Dawa [15].

All functions of the health system and public health policy are seriously reliant on the presence and use of quality HIS data [3, 22]. However, lack of quality data and poor usage are affecting the health system’s performance and the health of the society. This is evident by frequent over and under stocks of supplies, poor detection and management of outbreaks, and scarcity of human resources at different times [23].

Performance of Routine Information System Management (PRISM) framework categorized the factors that influence the data quality in to three groups; behavioral, technical, and organizational factors [24]. Level of knowledge [25], negligence, data manipulation for competition sake [26], motivation [27], and sense of responsibility [28] are among the behavioral factors associated with the data quality. User-friendliness of reporting format, and standardized indicators [29] are the technical factors affecting the data quality while availability of the training [30, 31], feedback [15], supervision [32], and data use [33, 34] are grouped under the organizational factors of the data quality.

Although there are studies conducted on the data quality, limited study has been conducted at the department level in this study area to explore the factors affecting the data quality. Moreover, the few studies conducted did not quantify the magnitude of the associations. Therefore, this study was aimed to assess the magnitude of the quality of routine health information system data and its determinants among public health facilities.

## Methods

### Study area and study period

The study was conducted in public health facilities of Harari regional State of Ethiopia from July 1 to 15, 2020. Located 518 km to the East of Addis Ababa, Harari Region is one of the ten regional States in Ethiopia with an estimated area of 311.25 km^2^. Based on the 2007 national census conducted by the Central Statistical Agency of Ethiopia (CSA), Harari Region has a total population of 183,415, and has 9 Districts (6 urban and 3 rural) and 36 kebeles (the smallest administrative units in Ethiopia) [35]. There are seven hospitals in the Harari Region of which one is owned by the Harari Regional Health Bureau while the rest are either other governmental or private hospitals. Among these, the 2 hospitals are governmental public health facilities. There are also 8 public health centers, 32 health posts, 10 not-for-profit private clinics, and 15 private clinics for profit in the Harari Region.

### Study design

A facility-based cross-sectional study design was employed.

### Study population

The study populations for this study were all departments that were implementing routine health management information systems (HMIS) in all public health facilities of Harari Regional State.

### Inclusion and exclusion criteria

#### Inclusion criteria

The study inclusion criterion was all health departments/units which were implementing routine health information system in the public health facilities of Harari regional state.

#### Exclusion criteria

Those departments/units that were closed during the data collection period were excluded from the study.

### Sample size determination and sampling procedure

The sample size of the study was determined by using a single population proportion formula

$${\text{n}}\, = \,\frac{{\mathop Z\nolimits_{{{\raise0.7ex\hbox{$a$} \!\mathord{\left/ {\vphantom {a 2}}\right.\kern-\nulldelimiterspace} \!\lower0.7ex\hbox{$2$}}}}^{2} p(1 - p)}}{{\mathop d\nolimits^{2} }}.$$

where n = Sample size, Zα/2 = Standard normal distribution corresponding to a significance level of alpha (α) of 0.05 = 1.96, P = magnitude of the data quality of routine health information system among departments in public health facilities of Dire Dawa (75.3%) [15] and d = degree of precision = 0.05. Accordingly

$${\text{n}} = \frac{{(1.96)^{2} * \left( {0.753} \right)*0.247 }}{{(0.05)^{2} }} + \, 10\% ,\;{\text{non - response}}\;{\text{rate}} = \, 314$$

Since the 245 total number of departments was less than 10,000, the correction formula was used and gave n_f_ = 314/1 + (314/245) = 138. However, since the existing departments implementing health information systems were found to be manageable, a census of all (245) departments found in all 42 public health facilities (8 health centers, 32 health posts, and 2 hospitals) was considered.

### Data collection instrument

The questionnaire was adapted from the Performance of Routine Information System Management assessment tool version 3.1 (see Additional file 1) [36], and used with little modifications to collect quantitative data. It comprised four sections: The first section was composed of questions related to socio-demographic characteristics of the department heads such as age, educational status, working experiences, professional category, salary, residence, and others. The second and third sections of the questionnaire included items assessing the technical, organizational, and behavioral factors associated with the quality of routine health information system data respectively. Observations, interviews, and document reviews guided by an observation checklist (fourth section of the questionnaire) were used to collect data on the departments’ data quality from all the departments through their respective department heads/representative of each department.

### Data collection procedures

Twelve health professionals who had basic data management training and prior experience of data collection and four health professionals who were members of the HIS monitoring team were assigned for the data collection and supervision respectively. Before the data collection, 2 days training was provided on the purpose, how to collect data, and on ethical issues emphasizing the importance of the safety of the participants, and data quality.

The data were collected by going to all the health facilities, explaining the aim of the study, ensuring the confidentiality of the data, obtaining the written consent from each facility head and participants, observing and interviewing to fill the checklist, and distributing the questionnaire to the department heads to read and fill the rest.

### Study variables

#### Dependent variable

Data quality was the dependent variable of the study.

#### Independent variables

The independent variables include:

*Organizational factors* training, feedback, supervision, computer, internet, reward, engagement in HIS activities, performance review meeting, and data use,

*Technical factors* presence of standard indicators, report formats, and trained person able to fill format, and.

*Behavioral factors* motivation, attitude, data manipulation for competition, negligence, sense of responsibility, knowledge, and data quality checking skills.

### Operational definitions

*Data quality* is an assessment of data's fitness to serve its purpose in a given context in-terms of accuracy, completeness and timeliness. For departments reporting on monthly basis, a real time data of 2 months (December 2019 and January 2020) were selected to assess data quality while for departments that make a report on quarterly basis, the first and third quarters of 2012 EFY (Ethiopian Fiscal Year) were selected to assess the data quality.

*Good quality data* The data that fits the criteria for the three quality dimensions—accuracy ≥ 80%, completeness ≥ 85%, and timeliness ≥ 85% [32, 37].

*Poor quality data* The data that does not fit the three criteria (accuracy < 80%, or completeness < 85%, or timeliness < 85%).

*Completeness* is the average of the source document or registration content completeness and report content completeness.

$${\text{Completenes}} = \frac{{\% \;{\text{of}}\;{\text{ register}}\;{\text{ content}}\;{\text{ completeness}} + \% \; {\text{of}}\;{\text{ report}}\; {\text{completeness}}}}{2}$$

The data is complete if the average is ≥ 85% [37].

*Register content completeness* was measured by dividing the number of completely recorded cases (taking the last 15 cases from the registration of the department for the selected month/quarter) by the total cases checked. If the total cases/entries are less than 15, the available cases are considered.

$${\text{register}}\;{\text{ content}}\;{\text{ completeness}}\% = \frac{{{\text{no}}{. }\;{\text{of}}\; {\text{completely}}\;{\text{ recorded}}\;{\text{ cases}}}}{{{\text{total}} \;{\text{cases}}}}*100$$

### Report content completeness

${\text{Report content completeness\% }}$ = $\frac{{{\text{ No}}.{\text{ of data elements reported in the report format}}}}{{\text{total number of expected data elements to be reported }}}{*}100$ [36]. For departments that do not keep the report copy with themselves, it was taken from the HMIS unit.

*Data Accuracy* was measured by recounting already reported data elements/indicators from the source document/register and compared with the one reported in the reporting format. The data elements/indicators for which the verification factor (recounted value from the source document divided by the value reported in the HMIS report) fell between 0.9 and 1.1 were regarded as accurate (have normal verification factor).

$${\text{Accuracy}} = \frac{{{\text{the}}\;{\text{ sum}}\;{\text{of}}\;{\text{accurate}}\;{\text{data}}\;{\text{elements}} \left( {{\text{recounted}}\;{\text{over}}\;{\text{reported}}\;{\text{ between}}\; 0.9 - 1.1} \right)}}{{{\text{total}}\;{\text{number}}\;{\text{of}}\;{\text{data}}\;{\text{elements}}\;{\text{checked}}}} *100$$

The department data is considered accurate if the average is ≥ 80% [32].

*Timeliness* was assessed as a report submission within the accepted time period through observing the reporting date on the reporting form of two randomly selected monthly reports. Departments at the health posts were expected to report from 20 to 22nd, departments at the health centers and hospitals report to the next level from 20 to 24th. The data of the department is timely if the average is ≥ 85% [37].

*Knowledge on HIS:* It was the knowledge of rationale of routine HIS data that was measured by using the three knowledge-related open-ended questions which have a total raw score of 7 and for which the answers were coded according to the themes on the PRISM assessment user guide [36]. The 50% mean score was used to classify the knowledge as good or poor.

### Data quality control

The pre-test of the questionnaire was done on 12 departments which are found in health facilities outside of the Harari Region to identify any ambiguity, consistency, and acceptability of the questionnaire as well as the time needed to fill the questionnaires. The necessary modifications were made before the actual data collection.

The quality of data was monitored frequently both in the field and during data entry. This was done in the field through close supervision of the data collectors. All completed questionnaires were examined for completeness and consistency during data collection. An incomplete and unclear filled questionnaire was given back to the study participants immediately.

### Data processing and analysis

Data were entered using Epi Data and exported to SPSS software version 25 for data recording, cleaning, and statistical analysis. Descriptive statics using frequencies, percentages, tables, and figures were used to describe the departments in the public health facilities, and the overall data quality was categorized as poor and good data quality. The figures in this study were free from apparent manipulation. Bivariate logistic regression analysis was done to identify variables that were candidates for multivariate analysis. All variables that have an association on bivariate analysis at a liberal *P* value of < 0.25 were considered for inclusion in the multivariate analysis. Afterwards, multivariate analysis was done to control the confounding effect of other variables and to identify independent predictors of routine health data quality in the health facilities. The magnitude and direction of the relationship between the variables were expressed as odds ratios (OR) with 95% CI and *P* value < 0.05 was used to declare the statistical significance. Model fitness was checked by using Hosmer–Lemeshow’s test at *P* value of > 0.05. The multicollinearity check was also carried out to detect the multicollinearity problem at a variance inflation factor (VIF) > 10. However, no multicollinearity problem was detected among the study independent variables.

## Results

### Description of the departments

From the total of 245 departments found in the 42 public health facilities of Harari region, 222 departments participated in the study with a 91% response rate. Among the 222 departments, 103 (46.39%), 82 (36.94%), and 37 (16.67%) were from the health posts, health centers, and hospitals respectively. Further, 42 (18.9%) maternal and child health, 17 (7.7%) Tuberculosis, and 6 (2.7%) Anti-retroviral therapy participated in the study (Table 1).

**Table 1:** Description of the departments participated in the study of quality of routine health information system data among departments in public health facilities of Harari Region, Ethiopia, 2020 (n = 222)

Departments	Frequency	Percent
Maternal and child health/MCH	42	18.9
< 5 out-patient department	40	18
Environmental health	25	11.3
Tuberculosis	17	7.7
Adult out-patient department	20	9
Pharmacy	10	4.5
Emergency	11	5
Laboratory	9	4.1
In-patient/wards	25	11.3
Voluntary counseling and testing (VCT)	7	3.2
Anti-retro viral therapy (ART)	6	2.7
Follow-up	2	0.9
Psychiatry	2	0.9
Critical intensive care unit (CICU)	1	0.45
Dental	1	0.45
Eye clinic	1	0.45
Neonate	1	0.45
Nutrition	1	0.45
Pathology	1	0.45
Total	222	100

### Socio-demographic characteristics of the department heads

The mean age of the respondents was 31.32 (± 6.226 SD) years with an average work experience of 8.65 (± 5.517 SD) years. About three quarters (74.3%) were females, more than half (51.8%) reside in urban areas, 64.4% were diploma holders, and 40.1% of the department heads were health extension workers (Table 2).

**Table 2:** Socio-demographic characteristics of the department heads participated in the study of quality of routine health information system data in public health facilities of Harari Region, Ethiopia, 2020 (n = 222)

Variables	Category	Frequency	Percent
Age category	20–24	11	5.0
	25–29	82	36.9
	30–34	82	36.9
	35–39	30	13.5
	40–44	5	2.3
	45–49	5	2.3
	50–54	3	1.4
	55–59	3	1.4
	60–64	1	0.5
Employment years	< 5	60	27
	5–9	81	36.5
	10–14	66	29.7
	≥ 15	15	6.8
Sex	Male	57	25.7
	Female	165	74.3
Residence	Rural	107	48.2
	Urban	115	51.8
Educational level	Diploma	143	64.4
	Bachelor degree	73	32.9
	Master degree	6	2.7
Professional category	Health extension worker	89	40.1
	Midwifery nurse	21	9.5
	Clinical nurse	74	33.3
	Others^a^	38	17.1

^a^Health officers, druggist, laboratory professionals, and Medical doctors

### Organizational factors

In this study, more than three quarters 172 (77.5%) of participants reported to have received supervision, and 33 (14.9%) of them have received refreshment trainings on HIS in the last 6 months (Table 3).

**Table 3:** Organizational factors affecting the quality of routine health information system data among departments in public health facilities of Harari Region, Ethiopia, 2020 (n = 222)

Variables	Categories	Frequency	Percent
Refreshment training in the last 6 months	Yes	33	14.9
	No	189	85.1
Feed-back	Received	137	61.7
	Not received	85	38.3
Supervision	Supervised	172	77.5
	Not supervised	50	22.5
Computer access	Yes	68	30.6
	No	154	69.4
Internet access	Yes	69	31
	No	153	69
Reward for good works	Present	59	26.6
	Absent	163	73.4
Engagement in HIS activities	Engaged	179	80.6
	Not engaged	43	19.4
Performance review meeting	Yes	183	82.4
	No	39	17.6
Presence of data use	Used	177	79.7
	Not used	45	20.3

### Technical factors

Most of the departments-183 (82.4%) have the standardized indicators, 178 (80.2%) reported that their reporting formats are user-friendly, and more than three quarters 174 (78.4%) have trained personnel able to fill the reporting formats.

### Behavioral factors

This study also revealed that majority of the department heads-216 (97.3%) were motivated to do HIS tasks, and more than two third (69.4%) have positive attitude towards HIS activities. In other ways, about 87 (39.2%) reported the presence of data manipulation in their departments, close to one third 70 (31.5%) reported presence of negligence, and only 48 (21.6%) had good knowledge of rationale of routine HIS data (Table 4).

**Table 4:** Behavioral factors influencing the quality of routine health information system data among departments in public health facilities of Harari Region, Ethiopia, 2020 (n = 222)

Variables	Categories	Frequency	Percent
Attitude towards HIS activities	Positive	154	69.4
	Negative	68	30.6
Motivation to do HIS tasks	Motivated	216	97.3
	Not motivated	6	2.7
Data manipulated	Yes	87	39.2
	No	135	60.8
Negligence in keeping data quality	Present	70	31.5
	Absent	152	68.5
Sense of responsibility in keeping data quality	Present	202	91
	Absent	20	9
Knowledge of rationale of routine HIS data	Good	48	21.6
	Poor	174	78.4
Data quality checking skill	Good	41	18.5
	Poor	181	81.5

### Level of the data quality

#### Data quality in-terms of accuracy

**Fig. 1:**
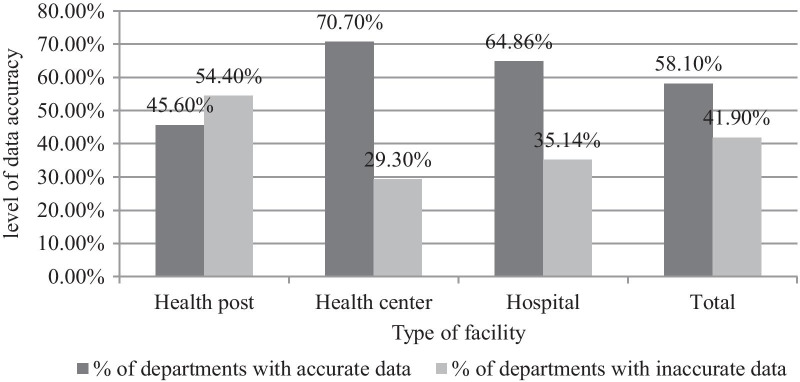
Level of data accuracy among departments found in different public health facility types of Harari Region, Ethiopia, 2020 (n = 222)

Among the 222 departments for which the data accuracy was checked, 129 (58.1%) of departments had accurate data, and the lowest proportion of accuracy (45.6%) was observed in the health posts (Fig. 1).

#### Data quality in-terms of completeness

**Fig. 2:**
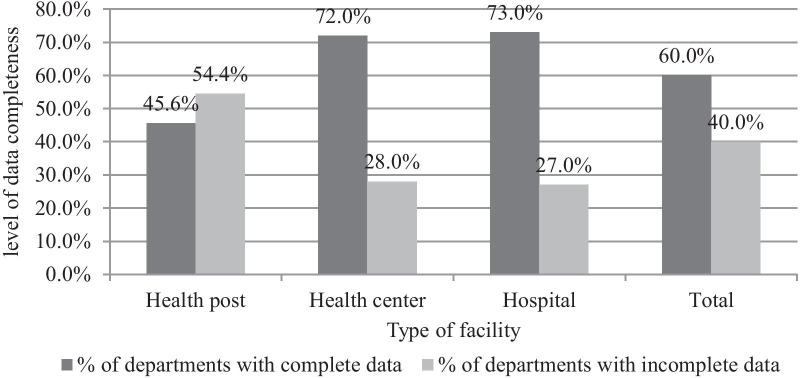
Level of completeness of data among departments in different types of public health facilities in Harari Region, Ethiopia, 2020 (n = 222)

Of the 17,589 data elements checked for report content completeness, 16,415 (93%) of the data elements were completely filled in the reporting format. Among the 5230 cases checked for registration content completeness with the relevant information, more than two third (69.6%) of the cases were completely registered on the registration. Overall, this study revealed that about 89 (40%) of the departments have incomplete data whereas the rest 133 (60%) have complete data (Fig. 2).

#### Data quality in-terms of timeliness

**Fig. 3:**
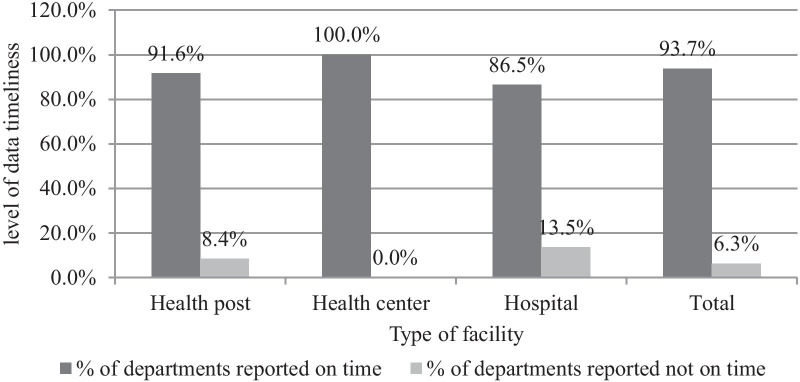
Level of data timeliness among departments in public health facilities of Harari Region, Ethiopia, 2020 (n = 222)

Our study also revealed that from the studied departments, 94 (91.26%) from the health posts, 82 (100%) from the health centers, and 32 (86.48%) departments from the hospitals submitted their report to the next level according to the national schedule. In general, the majority (93.7%) of the study units submitted their report on time while only 14 (6.3%) did not (Fig. 3).

#### Overall data quality

**Fig. 4:**
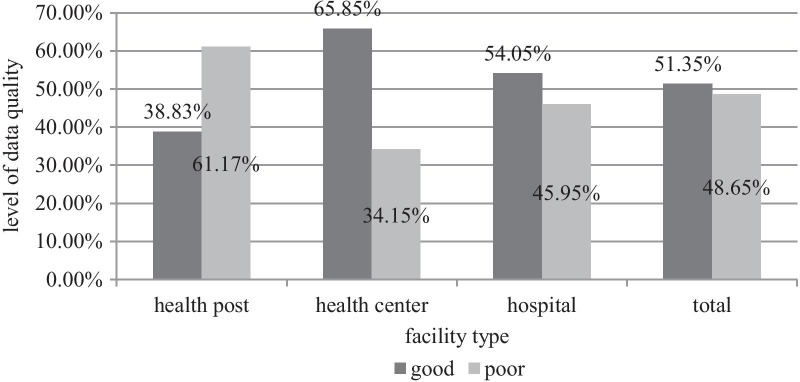
The level of overall data quality among departments found in different facility types of public health facilities in Harari Region, Ethiopia, 2020 (n = 222)

Of the total departments assessed, 114 (51.35%; 95% CI 44.6–58.1%) departments have good quality data. Moreover, more than one third-40 (38.83%), about two third 54-(65.85%), and more than half-(54.05%) of the departments at the health posts, health centers and hospitals respectively were found to have good quality data (Fig. 4).

**Fig. 5:**
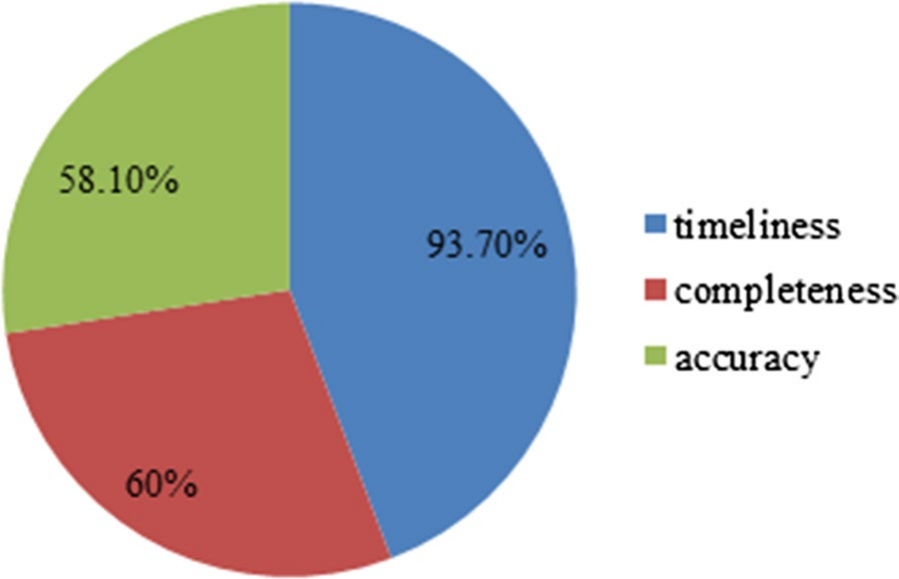
Accuracy, completeness and timeliness of Data among departments in public health facilities of Harari Region, 2020

Among the three data quality dimensions assessed in this study, timeliness of 93.7%, completeness of 60%, and accuracy level of 58.1% were observed among departments in the studied facilities (Fig. 5).

### Factors associated with quality of routine health information system data

In bivariate logistic regression, the level of education, residence, type of facility, standardized indicators, user-friendliness of reporting format, presence of trained person able to fill reporting formats, internet access, refreshment training, supervision and feedback were associated to the data quality. However, the type of facility, presence of trained person able to fill reporting formats and feed-back were significantly associated to the data quality in both bivariate and multivariate analysis. The departments that were found in the health centers were 2.5 times more likely to have good quality data than the departments found in the health posts (AOR = 2.499; 95% CI 1.059–5.897). The departments that have trained personnel able to fill the formats were 2.5 times more likely to have good quality data as compared to the departments that do not have the trained person (AOR = 2.474; 95% CI 1.124–5.445). The departments that received feed-back were 3 times more likely to have good quality data as compared to the departments that do not (AOR = 3.083; 95% CI 1.549–6.135) (Table 5).

**Table 5:** Factors associated to the quality of routine health information system data on logistic regression in public health facilities of Harari Region, Ethiopia, 2020 (n = 222)

Variables	Categories	Data quality		COR (95% CI)	AOR (95% CI)	*P* value
		Good	Poor			
Educational level	Degree and above	52	27	2.516 (1.422–4.452)	1.876 (0.755–4.661)	0.176
	Diploma	62	81	1^R^	1^R^	
Residence	Urban	69	43	2.318 (1.353–3.970)	1.547 (0.772–3.103)	0.219
	Rural	45	65	1^R^	1^R^	
Refreshment training	Yes	26	7	4.263 (1.764–10.30)	2.269 (0.825–6.237)	0.112
	No	88	101	1^R^	1^R^	
Standard indicators	Yes	101	82	2.463 (1.191–5.095)	1.273 (0.489–3.316)	0.622
	No	13	26	1^R^	1^R^	
Facility type	Health center	54	28	3.037 (1.660–5.559)	2.499 (1.059–5.897)*	0.037*
	Hospital	20	17	1.853 (0.868–3.955)	0.999 (0.310–3.222)	0.998
	Health post	40	63	1^R^	1^R^	
User friendly report format	Present	102	76	3.579 (1.730–7.404)	1.771 (0.682–4.599)	0.240
	Absent	12	32	1^R^	1^R^	
Trained person able to fill formats	Present	99	75	2.904 (1.471–5.733)	2.474 (1.124–5.445)*	0.024*
	Absent	70	83	1^R^	1^R^	
Feed-back	Received	86	51	3.433 (1.942–6.068)	3.083 (1.549–6.135)*	0.001*
	Not received	28	57	1^R^	1^R^	
Supervision	Received	96	76	2.247 (1.171–4.307)	1.351 (0.620–2.943)	0.450
	Not received	18	32	1^R^	1^R^	
Internet access	Yes	44	25	2.087 (1.163–3.746)	0.610 (0.268–1.388)	0.238
	No	70	83	1^R^	1^R^	

*COR* crude odds ratio, *AOR* adjusted odds ratio, *1*^*R*^ reference category

*P value < 0.05 from multivariate analysis

## Discussion

This study provides an insight into the various technical, behavioral, and organizational factors that influence quality of routine health data. The accuracy of data in this study was found to be 129 (58.1%) and it was less than the accuracy of data reported from Hadiya zone, Southern Ethiopia where seventy six percent of the departments at the health center had accurate data [17] and 79% in Nigeria [38]. The difference might be because of the difference in the type of facilities and level of the feedback provided to the departments in which 95.8% of the departments at Hadiya zone [17] and 61.7% of the departments in this study received the feedback. Also, the interval of verification factor used to measure the data accuracy in Nigeria was wider (0.85–1.15) [38] than the verification factor interval used in this study (0.9–1.1) to measure the data accuracy. Generally, data accuracy may be affected by errors that occur during data entry, intentionally manipulating the data for different reasons possibly competition among the staffs and facilities, false report to increase achievement, and reports not made on time. The study conducted in Tanzania supports some of these explanations; for example, data manipulation can affect the accuracy of data [39].

In this study, the 69.6% registration (source document) content completeness was lower than the 93% report content completeness. This is supported by the recently published study which was conducted in East Wollega where the 78.2% registration content completeness was less than the 86% report content completeness indicating that the health workers focus more on managing patients rather than recording data due to the work load and lack of commitment to the data [40].

The 93.7 percent timeliness of the data revealed in this study was closer to the one reported in the data quality review conducted by the Ethiopian public health institute which was 100% data timeliness in Harari Region [18] but higher than the timeliness reported from the other parts of Ethiopia-70% in East Wollega and 89% in West Wollega [41]. The easy accessibility of the health facilities in our study area is the possible explanation for the difference observed.

The result of the study revealed that more than half (51.35%) of the departments implementing routine health information system have good levels of data quality. This is similar with the findings from many developing countries that the data quality falls between 34 and 72% [9]. However, it is lower than the result from the studies conducted in Dire Dawa and Addis Ababa which reported three fourth (75.3%), [15] and 76.22% [21] level of good quality data respectively. This might be because of the difference in the way the dimensions of the data quality were measured. The study conducted in Dire Dawa measured the completeness in-terms of the report completeness only while in this study the completeness was measured in-terms of both the registration content completeness and report content completeness. The difference might also be attributed to the effect of Corona Virus Disease (COVID-19) on the health information system performance including data quality. Because this study was conducted while the COVID-19 was seriously challenging the health system as in general.

The departments that were found in the health centers were 2.5 times more likely to have good quality data compared to those found in the health posts. This is evident by the findings from the pioneering regions of Ethiopia in which the data quality was better at the health centers and hospitals than at the health posts [42]. The low level of education among the staffs at the health posts (all are diploma holder and below), the larger amount of data collected by limited number of health extension workers and lack of HMIS personnel who closely monitor the data quality as compared to the health centers and hospitals are the possible reasons for the variation. It might also be due to the more attention given by the government and other stakeholders such as Capacity Building and Mentorship Program (CBMP), a program supporting the health centers, and hospitals through HMIS trainings and onsite mentorships.

This study found that presence of the trained personnel able to fill the reporting formats, and the provisions of feed-backs were significantly associated with the good quality data. This was supported by the study conducted in Dire Dawa where the presences of trained staffs and feed-back were significantly associated to the data quality with AOR = 2.25; 95% CI 1.082–4.692 and AOR = 2.48; 95% CI 1.262–4.846 respectively [15]. A recent scoping review conducted also showed that the combination of feed-back with the other capacity building activities contribute to the data quality improvement [43]. Training can make clarity on the issues of HIS related activities and tools and increases familiarity with the HIS tools such as registers, reporting formats and information communication technology soft wares.

Although supportive supervision showed association with the data quality on bivariate logistic regression, it was not significantly associated to the data quality on multivariate logistic regression in this study. This was different from the finding of the study conducted in Gurage Zone in which the supervision was associated to the community health information system performance (data quality and use) [32]. The difference might be attributed to the quality of supervision as noted from the study in Tanzania [25]. The other possible justification is that in most practical cases, supervision is just to find fault rather than being supportive supervision. But, it is the targeted supportive supervision which helps the departments to fill their gap in data recording, processing, analyzing, reporting and data quality checking.

The limitation of this study was its inability to show the consistency between the data in the routine health information system and that same data in the real-world since the study addressed only the three dimensions of data quality. Future studies should incorporate qualitative studies to have a deeper insight on the behavioral factors that influence data quality.

## Conclusion

The level of good data quality among the departments in the public health facilities of Harari region was less than the 80% expected national level. The refreshment training given to the staff was found to be low. The type of facility, lack of trained personnel able to fill the reporting formats, and the feedback were the factors that significantly associated with the data quality. Continuous refreshment in-service HMIS related training should be arranged and provided by Harari Regional health bureau and other stakeholders to increase the knowledge and skills of the health workers. It is also better for the supervisors at different levels of the Harari region particularly woreda health offices to provide supportive supervision focusing on the data quality and provide feedback to the departments regularly.

## Supplementary Information

**Additional file 1**. English, Amharic and Afan Oromo versions of the questionnaire.

## Data Availability

The data sets used and/or analyzed during the current study are available from the corresponding author on reasonable request.

## Abbreviations

HIS: Health information system
HMIS: Health management information system
PRISM: Performance of Routine Information System Management
WHO: World Health Organization

## References

[1] World Health Organization (WHO). Everybody's business: strengthening health systems to improve health outcomes: WHO’s FrameWork for Action. 2007. https://www.who.int/healthsystems/strategy/everybodys_business.pdf. Accessed 1 Nov 2019.

[2] Measure Evaluation. Routine health information systems: a curriculum on basic concepts and practice, facilitators' guide. 2017. https://www.measureevaluation.org/resources/publications/sr-16-135b. Accessed 24 Nov 2019.

[3] Ministry of Health (MOH). HMIS information use guide technical standards area 4: version 2. HMIS scale-up project. Federal Democratic Republic of Ethiopia. 2013. https://www.measureevaluation.org/resources/publications/ms-13-70. Accessed 30 Oct 2019.

[4] Yarinbab TE, Assefa MK. Utilization of HMIS data and its determinants at health facilities in East Wollega Zone, Oromia Regional State, Ethiopia: a health facility based cross-sectional study. J Med Health Sci. 2018;7(1):4–9.

[5] Ministry of Health (MOH). HSTP (health sector transformation plan): 2015/16–2019/20 (2008–2012 EFY). 2015. https://ehia.gov.et/sites/default/files/Resources/HSTP%20Final%20Print%202015-11-27%20Print%20size.pdf. Accessed 28 Feb 2017.

[6] Drobac PC, Basinga P, Condo J, Farmer PE, Finnegan KE, Hamon JK (2013). Comprehensive and integrated district health systems strengthening: the Rwanda Population Health Implementation and Training (PHIT) Partnership. BMC Health Serv Res.

[7] Kumar M, Gotz D. System design barriers to HIS data use in low and middle-income countries: a literature review. UNC SILS Technical Report 2016-01. 2016. https://sils.unc.edu/sites/default/files/general/research/UNCSILS-TR-2016-01.pdf. Accessed 21 Oct 2019.

[8] Lippeveld T (2017). Routine health facility and community information systems: creating an information use culture. Glob Health Sci Pract.

[9] Belay H, Lippeveld T. Inventory of PRISM framework and tools: application of PRISM tools and interventions for strengthening routine health information system performance. Chapel Hill, NC: MEASURE Evaluation. 2013. https://www.measureevaluation.org/resources/publications/wp-13–138. Accessed 3 Jan 2020.

[10] Rowe AK (2009). Potential of integrated continuous surveys and quality management to support monitoring, evaluation, and the scale-up of health interventions in developing countries. Am J Trop Med Hyg.

[11] Mate KS, Bennett B, Mphatswe W, Barker P, Rollins N (2009). Challenges for routine health system data management in a large public programme to prevent mother-to-child HIV transmission in South Africa. PLoS ONE.

[12] Mutale W, Chintu N, Amoroso C, Awoonor-Williams K, Phillips J, Baynes C (2013). Improving health information systems for decision making across five sub-Saharan African countries: implementation strategies from the African Health Initiative. BMC Health Serv Res.

[13] Mbondji PE, Kebede D, Soumbey-Alley EW, Zielinski C, Kouvividila W, Lusamba-Dikassa P-S (2014). Health information systems in Africa: descriptive analysis of data sources, information products and health statistics. J R Soc Med.

[14] Adane A, Adege TM, Ahmed MM, Anteneh HA, Ayalew ES, Berhanu D (2021). Routine health management information system data in Ethiopia: consistency, trends, and challenges. Glob Health Action.

[15] Teklegiorgis K, Tadesse K, Mirutse G, Terefe W (2016). Level of data quality from health management information systems in a resources limited setting and its associated factors, Eastern Ethiopia’. S Afr J Inf Manag.

[16] Kidane T, Ejigu G, Girma T (2014). Assessment of health management information system implementation in Ayder Referral Hospital, Mekelle, Ethiopia. Int J Intell Inf Syst.

[17] Abera E, Daniel K, Letta T, Tsegaw D (2016). Utilization of health management information system and associated factors in Hadiya zone health centers, Southern Ethiopia. Res Health Sci.

[18] Ethiopia Public Health Institute (EPHI). Health data quality review: system assessment and data verification for selected indicators. 2018. https://www.ephi.gov.et/images/pictures/download_2011/Ethiopia-Data-Quality-Review-DQR-report--2018.pdf. Accessed 21 Oct 2019.

[19] Ouedraogo M, Kurji J, Abebe L, Labonte R, Morankar S, Bedru KH (2019). A quality assessment of health management information system (HMIS) data for maternal and child health in Jimma Zone, Ethiopia. PLoS ONE.

[20] Haftu B, Taye G, Ayele W, Habtamu T, Biruk E. A mixed-methods assessment of routine health information system (RHIS) data quality and factors affecting it, Addis Ababa City administration, Ethiopia, 2020. Ethiop J Health Dev. 2020;35(SI-1):15–24.

[21] Ayele W, Biruk E, Habtamu T, Taye G, Tamire M, Addissie A (2021). Data quality and it’s correlation with routine health information system structure and input at public health centers in Addis Ababa, Ethiopia. Ethiop J Health Dev.

[22] World Health Organization (WHO). Improving data quality: a guide for developing countries. 2003. https://apps.who.int/iris/handle/10665/206974. Accessed 1 Nov 2019.

[23] Bram JT, Warwick-Clark B, Obeysekare E, Mehta K (2015). Utilization and monetization of healthcare data in developing countries. Big Data.

[24] Aqil A, Lippeveld T, Hozumi D (2009). PRISM framework: a paradigm shift for designing, strengthening and evaluating routine health information systems. Health Policy Plan.

[25] Simba DO, Mwangu MA (2006). Factors Influencing Quality Of Health Management Information System (HMIS) data the case of Kinondoni District in Dares Salaam Region, Tanzania. East Afr J Public Heath.

[26] Misganu E, Abraham A, Emebet M, Sinafikish A, Temesgen K, Mekonnen S (2019). Understanding performance data: health management information system data accuracy in southern nations nationalities and people’s region, Ethiopia. BMC Health Serv Res.

[27] Mucee EM, Odhiambo-Otieno GW, Kaburi LW, Kinyamu RK (2016). Routine health management information use in the public health sector in Tharaka Nithi County, Kenya. Imperial J Interdiscip Res (IJIR).

[28] MeasureEvaluation. Tools for data demand and use in the health sector: performance of routine information system management (PRISM) tools. 2011. https://www.measureevaluation.org/resources/publications/ms-11-46-d.

[29] Silas NK. Factors influencing performance of routine health information system: the case of Garissa Subcounty, Kenya. A master’s Thesis, University of Nairobi. 2017.

[30] Ahanhanzo YG, Ouedraogo LT, Kpozèhouen A, Coppieters Y, Makoutodé M, Wilmet-Dramaix M (2014). Factors associated with data quality in the routine health information system of Benin. Arch Public Health.

[31] Nwankwo B, Sambo MN (2018). Can training of health care workers improve data management practice in health management information systems: a case study of primary health care facilities in Kaduna State, Nigeria. Pan Afr Med J.

[32] Tsedeke M. Community health management information system performance and factors associated with at health post of Gurage Zone, SNNPR, Ethiopia. A Master’s Thesis, University Of Gondar and Addis Continental Institute of Public Health. 2015. https://www.researchgate.net/profile/Tsedeke_Mathewos/publication/304567082_Community_health_management_information_system_Performance_and_factors_associated_with_at_health_post_of_Gurage_zone_SNNPR_Ethiopia/links/5773798508aeef01a0b66949/Community-health.

[33] Orr K (1998). Data quality and systems theory. Commun ACM.

[34] Braa J, Heywood A, Sahay S (2012). Improving quality and use of data through data-use workshops: Zanzibar, United Republic of Tanzania. Bull World Health Organ.

[35] Central Statistical Agency (CSA). Population and housing census of Ethiopia. 2007. https://www.statsethiopia.gov.et/census-2007-2/.

[36] Aqil A, Lippeveld T, Moussa T, Barry A. PRISM (performance of routine information system management) tools user guide. 2012. www.cpc.unc.edu/measure.

[37] Gebrekidan M, Hajira M, Habtamu T, Negusu W, Dereje M, Fatoumata N-T. Data quality and information use: asystematic review to improve evidence in Ethiopia. Afr Health Monit. 2012;14:53–60.

[38] Adejumo A, Mathews V. An assessment of data quality in routine health information systems in Oyo State, Nigeria. A master’s Thesis, University of the Western Cape. 2017. http://hdl.handle.net/11394/5497.

[39] Rumisha SF, Lyimo EP, Mremi IR, Tungu PK, Mwingira VS, Mbata D (2020). Data quality of the routine health management information system at the primary healthcare facility and district levels in Tanzania. BMC Med Inform Decis Mak.

[40] Kebede M, Adeba E, Chego M (2020). Evaluation of quality and use of health management information system in primary health care units of east Wollega zone, Oromia regional state, Ethiopia. BMC Med Inform Decis Mak.

[41] Fikru ND, Dereje BD. Evaluation of HMIS data quality and information use improvement for local action-oriented performance monitoring in Beghi District in West Wollega, Oromia, Ethiopia. J Health Med Nurs 2018;50:2422–8419.

[42] Woldemariam H, Habtamu T, Fekadu N, Habtamu A (2010). Implementation of an integrated health management information system and monitoring and evaluation (HMIS/M&E) system in Ethiopia: progress and lessons from pioneering regions. Q Health Bull.

[43] Lemma S, Janson A, Persson L-Å, Wickremasinghe D, Källestål C (2020). Improving quality and use of routine health information system data in low- and middleincome countries: a scoping review. PLoS ONE.

