# Supplementary material for: Assessment of quality of routine health information system data and associated factors among departments in public health facilities of Harari region, Ethiopia
Source: BMC Med Inform Decis Mak. 2021 Oct 19;21:287. doi: 10.1186/s12911-021-01651-2 (PMC8524221; doi:10.1186/s12911-021-01651-2)
Supplement: Supplementary file 1 — Additional file 1. English, Amharic and Afan Oromo versions of the questionnaire. [file 12911_2021_1651_MOESM1_ESM.pdf]

1. English version data collection questionnaires

Respondent ID: ___ Data collection date __________Data collection starting time_____

Introduction

This is an independent study, to improve Management Information Systems in the health sector. The objective of this survey is to help develop interventions for improving information system and data quality. Please express your opinion honestly. Your responses will remain confidential and will not be shared with anyone, except for presented table forms. We appreciate your assistance and co-operation in completing this study.

Thank you.

Part I. Questions related to socio-demographic characteristics

ID1.	Name of facility_____________ ID2.Unit/department name_________________		
ID3.	District_________________________		
ID4.	Type of facility:	1. health post 2.health center 3.hospital	
DD1.	Title of the person filling the questionnaire (circle answer):	1. OPD case team leader 2. Pharmacy head 3. Health facility manager 4.Ward head 5. HMIS officer 6. Laboratory team leader 7.Other facility Staff (specify____________)	
DD2.	Age---------------------		
DD3	Years of employment ----------------------		
DD4.	Salary per month_______________________ETB		
DD5.	Sex:	1. Male 2.Female	
DD6.	Residence of the respondent:	0. Rural 1. Urban	
DD7.	Educational status:	1. Diploma 2. Bachelor Degree 3. Master's Degree 4. PhD or doctorate<br>5. Others (specify______________)	
DD8.	Professional category:	1. Health Extension Worker 2. Midwifery Nurse 3. Clinical Nurse 4. Environmental Health 5. Pharmacist/druggist 6. Laboratory professional 7. HO/Health Officer 8. MD/Doctor 9. Occupational health 10.Nutrition 11. Psychiatry 12. Health informatics 13. Radiology professional 14. If other specify_________________	

We would like to know your opinion about how strongly you agree with certain activities carried out by you and your organization. There are no right or wrong answers, but only expression of your opinion on a scale. The scale is about assessing the intensity of your belief and ranges from strongly disagree (1) to strongly agree (7). You have to determine first whether you agree or disagree with the statement. Second decide about the intensity of agreement or disagreement. If you disagree with statement then use left side of the scale and determine how much disagreement that is – strongly disagree (1), somewhat disagree (2), or disagree (3) and circle the appropriate

answer. If you are not sure of the intensity of belief or think that you neither disagree nor agree, then circle 4. If you agree with the statement, then use right side of the scale and determine how much agreement that is – agree (5), somewhat agree (6), or strongly agree (7) and circle the appropriate answer. Please note that you might agree or disagree with all the statements and similarly you might not have the same intensity of agreement or disagreement and thus variations are expected in expressing your agreement or disagreement. We encourage you to express those variations in your beliefs. Please be frank and choose your answer honestly. To what extent, do you agree with the following on a scale of 1-7?

Part II. Assessment of technical factors

1. Strongly Disagree 2. Somewhat Disagree 3. Disagree 4. Neither Disagree nor Agree 5. Agree 6. Somewhat Agree 7. Strongly Agree								
S. No.	Questions							
1	There is standardized set of indicator.	1	2	3	4	5	6	7
2	There is friendly format for reporting result.	1	2	3	4	5	6	7
3	Do you agree that there is trained staff able to fill out format?	1	2	3	4	5	6	7

Part III. Organizational and Behavioral Assessment Tool

1. Is there anyone from the department who received training in HMIS related activities in the last six months? 0. No 1.Yes								
2. Staff can access computer 0. No 1.Yes, observed								
3. Staff can access internet 0. No 1.Yes								
1. Strongly Disagree 2. Somewhat Disagree 3. Disagree 4. Neither Disagree nor Agree 5. Agree 6. Somewhat Agree 7. Strongly Agree								
S. No.	Questions							
S1.	Superiors provide regular feedback to their staff through regular report based on evidence	1	2	3	4	5	6	7
S2.	Superiors emphasize data quality in monthly reports	1	2	3	4	5	6	7
S3.	Superiors provide supportive supervision to the staff focusing on data quality	1	2	3	4	5	6	7
S4.	Superiors check data quality at the facility and higher level regularly	1	2	3	4	5	6	7
S5.	Superiors report on data accuracy regularly	1	2	3	4	5	6	7
P3.	Staffs are rewarded for good work.	1	2	3	4	5	6	7
A1	Staffs perceive data collection as a useless	1	2	3	4	5	6	7

.	activity.							
A2.	Staffs perceive data collection as waste of care provider's time	1	2	3	4	5	6	7
E1	Staffs engage actively in all activities of the health information system.	1	2	3	4	5	6	7
PR.	Health facility conducts performance review meeting/self-assessment with concerned bodies.	1	2	3	4	5	6	7
U1	Staffs use HMIS data for day to day management of the facility	1	2	3	4	5	6	7
U2	Staffs display data for monitoring their set target by means of graphs and tables.	1	2	3	4	5	6	7
U3	Staffs use HMIS data to identify and manage epidemics	1	2	3	4	5	6	7
U4	Staffs use HMIS data for Drug supply and management:	1	2	3	4	5	6	7
U5	Staffs use HMIS data for community education and mobilization	1	2	3	4	5	6	7
B1.	Collecting information which is not used for decision making discourages me.	1	2	3	4	5	6	7
B2.	Collecting information makes me feel bored	1	2	3	4	5	6	7
B3.	Collecting information is meaningful for me:	1	2	3	4	5	6	7
B4.	Collecting information gives me the feeling that data is needed for monitoring facility performance:	1	2	3	4	5	6	7
B5.	Collecting information gives me the feeling that it is forced on me:	1	2	3	4	5	6	7
B6.	Collecting information is appreciated by co-workers and superiors:	1	2	3	4	5	6	7
B7.	Staffs manipulate the data for the sake of competition	1	2	3	4	5	6	7
B8.	I am negligent in keeping data quality.	1	2	3	4	5	6	7
Ac1	Staffs feel guilty for not accomplishing the set target/performance:	1	2	3	4	5	6	7
Ac2	Staffs are made accountable for poor performance:	1	2	3	4	5	6	7
R1	Staffs are punctual	1	2	3	4	5	6	7
R2	Staffs feel committed in improving health status of the target population:	1	2	3	4	5	6	7

R3	Staffs document their activities and keep records	1	2	3	4	5	6	7

K1.Describe at least three reasons for collecting data on monthly basis on the followings:
K1A.Diseases
1.
2.
3.
K1B.Immunization
1.
2.
3.
3.
K2. Why is population data of the target area needed?
1.
2.
3. CS. Describe at least three ways of checking data quality.

1.
2.
3.
Name of data collector: ________________________Signature:___________
Professions of data collector____________________
Address of data collector: ___________________
Date of data collection: ________________
Time at end of finish data collection: ____________
Total time taken to collect data:______________
Thank you!

2. Amharic version data collection questionnaires

መግብያ

ይህ ጥናት እራሱን የቻለ ሲሆን በጤናዉ ሴክተር የመረጃ ስርአት አስተዳደርን ለማሻሻል ነዉ:: የጥናቱ አላማ ለመረጃ ስርአት ማሻሻልና ለማስረጃ ጥራት የሚወሰዱ እርምጃዎችን ለማገዝ ነዉ:: እባኮን ሃሳባችሁን በታማኝነት ግለጹልን::የመልስዎ ሚስጢር የሚጠበቅና በሰሌዳ መልክ ከመቅረብ ዉጪ ለማንም የማይካፈልነዉ::ይህን ጥናት ለመሙላት ያደረጋችሁትን ድጋፍና ተብብር እናበረታታለን::

እናመሰግናለን!

ክፍል 1-ከህብረተሰብ ዲሞግራፊ ጋር የተያያዙ ጥያቄዎች

ID1.		የተቋሙ፣ስም-------------------- ID2.የክፍሉ/ድፓርትመንቱ ስም___________________________________	
ID3.		ወረዳ------------------	

ID4.		የተቋሙ ፣አይነት	1. ጤና ኬላ 2. ጤና ጣቢያ 3. ሆስፒታል	
DD1.		መጠይቁን የሚሞላ፣የ ሰዉ ፣ርእስ (መልሱን ክበበዉ)	1. የተመላላሽ፣ህክምና፣ቡድን ፣መሪ 2. የ መድሃኒት፣ክፍል፣ማሪ 3. የጤና፣ተቋሙ ፣አስተዳዳሪ 4. የ ዋርድ(Ward) መሪ 5. የ HMIS መኮንን 6. የላቦራቶሪ ፣ቡድን፣መሪ 7. ሌላ ካለ፣ ይስዩ---------	
DD2.		እድሜ--------------		
DD3		የአገልግሎት ፣አመት---------		
DD4.		ወራዊ ደሞስ-------------በ ኢትዮጵያ ብር		
DD5.		ጾታ	1. ወንድ 2. ሴት	
DD6.		የመላሹ መኖርያ	1. ገጠር 2. ከተማ	
DD7.		የትምህርት፣ደረጃ	1.ድፕሎማ 2. የመጀመርያ፣ድግሪ 3. ማስቴርስ ፣ድግሪ 4. ዶክትሬት ድግሪ 5. ሌላ ካለ ይስዩ-------------	
DD8.		የሙያ መደብ	1. የጤና፣ኤክስቴንሽን፣ ሰራተኛ 2. አዋላጅ፣ ነርስ 3. ክሊንካል፣ ነርስ 4. የአካባቢ፣ጤና 5. የመድሃኒት፣ባለሙያ 6. የላቦራቶሪ፣ባለሙያዘ 7. ጤና፣መኮንን 8. ሀኪም 9.የስራ ቦታ ጤና (occupational health) 10. ስነ፣ምግብ 11. ሳይካትሪ 12. የጤና፣መረጃ 13. የራድዮሎጂ ባለሙያ 14. ሌላ፣ካለ፣ይስዩ-------	

በእርስዎና በድርጅታችሁ ስለሚከናወኑ የተወሰኑ ስራዎች በምንያህል ጥንካሬ እንደሚስማሙ ሃሳብዎን ለማወቅ እንወዳለን:: ሃሳብዎን በተቀመጠ እስኬል ላይ ከመግለጽ ባለፈ ትክክል ወይም ስህተት የሚል መልስ የለም:: እስኬሉ የእርስዎ አመኔታ ጥንካሬን ስለመዳሰስ ሲሆን በጠንካራ አልስማማበትም (1) እስከ በጠንካራ እስማማበታለሁኝ (7) የሚሄድ ነዉ:: በመጀመርያ ደረጃ ከአባባሉ ጋር መስማማት አለመስማማትዎን መለየት ነዉ:: ከዛ ስለመስማማት አለመስማማትዎን ጥንካሬ መወሰን ነዉ:: ከአባባሉ ጋር የማይስማሙ ከሆነ በግራ በኩል ያለዉን እስኬል ይጠቀሙና በምንያህል እንደማይስማሙ ማለትም በጠንካራ አልስማማበትም(1) የተወሰነ አልስማማበትም(2) ወይም አልስማማበትም(3) ብሎ የሚሆነዉን መልስ ያክብቡ:: ስለ አመኔታዉ ጥንካሬ እርግጥ ካልሆኑ ወይም ደግሞ ስለ አለመስማማትና ስለመስማማት ሃሳብ ከሌለ 4ን ይምረጡ:: ከአባባሉ ጋር የሚስማሙ ከሆነ ደግሞ ከእስኬሉ በቀኝ ጎን ያለዉን ይጠቀሙና በምንያህል እንደሚስማሙ ማለትም እስማማበታለሁኝ(5) የተወሰነ እስማማበታለሁኝ(6) በጠንካራ እስማማበታለሁኝ(7) ብሎ መልሱን ያክብቡ:: ልብ በሉ! ከሁሉም አባባሎች ጋር መስማማትም ሆነ አለመስማማት እንደሚቻል ሁሉ ተመሳሳይ የመስማማት ና ያለመስማማት ጥንካሬ ሳይኖር ስለሚችል መስማማት አለመስማማትዎን ሲገልጹ ልዩነቶች ይጠበቃሉ:: እነዛ ልዩነቶችንም እንዲገልጹልን እናበረታታለን:: እባኮን መልሱን በታማኝነት ይምረጡ:: ከ1-7 እስኬል ምንያህል ከዚህ በታች ካሉ ጋር ይስማማሉ ?

ክፍል-2 የቴክንክ ምክንያቶች ዳሰሳ

1. በጠንካራ አልስማማበትም 2. የተወሰነ አልስማማበትም 3. አልስማማበትመ 4.መስማማትም ፣ሆነ አለመስማማት የለኝም 5. እስማማበታለሁኝ 6. የተወሰነ እስማማበተለሁኝ 7. በጠንካራ እስማማበታለሁኝ

ተ.ቁ	ጥያቄዎች							
1	እስታንዳርድ/ልከኛ ፣የሆነ፣ጠቋሚ(standardized indicator) አለ::	1	2	3	4	5	6	7
2	ዉጤትን፣ሪፖርት፣ለማድረግ፣ቀላል፣የሆነ፣የሪፖርት፣ቅጽ፣አለ	1	2	3	4	5	6	7
3	ቅጹን ፣መሙላት ፣የሚችል ፣የሰለጠነ ፣እስታፍ ፣አለ::	1	2	3	4	5	6	7

ክፍል-3 የድርጅት ና የባህሪ ዳሰሳ መሳርያ

1. ከዚህ ድፓርትሜንት ባለፉት ስድስ ወራት ዉስጥ ከHMIS ጋር የተያያዘ ስልጠና የወሰደ አለ? 0. አይደለም1. አዎ								
2. ሰራተኞች ኮምፒዩተርን ማግኘት ይችላሉ 0. አይደለም1. አዎ፣ ታይቶአል								
3. ሰራተኞች ኢንተርኔትን ማግኘት ይችላሉ 0. አይደለም1. አዎ								
1. በጠንካራ አልስማማበትም 2. የተወሰነ አልስማማበትም 3. አልስማማበትመ 4.መስማማትም ፣ሆነ አለመስማማት የለኝም 5. እስማማበታለሁኝ 6. የተወሰነ እስማማበተለሁኝ 7. በጠንካራ እስማማበታለሁኝ								
ተ.ቁ	ጥያቄዎች							
S 1	ከላይ ያሉ ሰዎች በቋሚ ሪፖርት በኩል ማስረጃ ላይ ተመሰርቶ በቋሚነት ግብረ-መልስ ይሰጣሉ::	1	2	3	4	5	6	7
S 2	ከላይ ያሉ ሰዎች በወራዊ ዘገባ ዉስጥ ለሚገኘዉ ለማስረጃ ጥራት ትኩረት ይሰጣሉ	1	2	3	4	5	6	7
S 3	ከላይ ያሉ ሰዎች በእስታንዳርዱ መሰረት ለሰራተኞች የድጋፍ ሱፔርቪዥንን ያደርጋሉ	1	2	3	4	5	6	7
S 4	ከላይ ያሉ ሰዎች በተቋምና ከዛ በላይ ባለዉ ደረጃ የማስረጃ ጥራትን ያያሉ::	1	2	3	4	5	6	7
S 5	ከላይ ያሉ ሰዎች ስለ ማስረጃ ጥራትበየጊዜዉ ይዘግባሉ	1	2	3	4	5	6	7
P 3	ሰራተኞች ለሰሩት መልካም ስራ ይሸለማሉ	1	2	3	4	5	6	7
A 1	ማስረጃ ማሰባሰቡ እንደማይረባ ስራ ሆኖ በእስታፍ ይሰማቻቃል	1	2	3	4	5	6	7
A 2	ማስረጃ ማሰባሰቡ የሰራተኞችን ጊዜ እንደማጥፋት ሆኖ በእስታፍ ይሰማቻቃል	1	2	3	4	5	6	7

E 1	ሰረተኞች በተቋሙ ዉስጥ ስሚደረገዉ ስራ ሁሉ በንቃት ይሳተፋሉ	1	2	3	4	5	6	7
P R	የጤና ተቋሙ ጉዳዩ ከሚመለከታቸዉ አካላት ጋር የአፈጻጸም ግምገማን ያደርጋል	1	2	3	4	5	6	7
U 1	ሰረተኞች ለእለት እለት የተቋሙ መስተዳድር የHMIS ማስረጃን ይጠቀማሉ	1	2	3	4	5	6	7
U 2	ሰራተኞች የተቀመጠዉን ግብ ስመከታተል በግራፍ ና በሰሌዳ ማስረጃን ያሳያሉ	1	2	3	4	5	6	7
U 3	ሰራተኞች ወረርሽኝን ስመለየትና ስመቆጣጠር የHMIS ማስረጃን ይጠቀማሉ	1	2	3	4	5	6	7
U 4	ሰራተኞች ስመድሃኒት አቅርቦትና ቁጥጥር የHMIS ማስረጃን ይጠቀማሉ	1	2	3	4	5	6	7
U 5	ሰራተኞች ህብረተሰቡን ስማስተማር ና ስማነሳሳት የHMIS ማስረጃን ይጠቀማሉ	1	2	3	4	5	6	7
B 1	ስዉሳኔ የማይዉል መረጃን ማሰባሰብ ያስጠላኛል	1	2	3	4	5	6	7
B 2	መረጃን ማሰባሰብ ይሰለቸኛል	1	2	3	4	5	6	7
B 3	መረጃን ማሰባሰብ ስኔ ትርጉም አለዉ	1	2	3	4	5	6	7
B 4	መረጃን ማሰባሰብ የተቋሙ አፈጻጸምን ስመከታተል ማስረጃ እንደሚያስፈልግ ስሜት ይሰጠኛል	1	2	3	4	5	6	7
B 5	መረጃን ማሰባሰብ በግድ እንደተጫነብኝ ነገር ሆኖ ይሰማኛል	1	2	3	4	5	6	7
B 6	መረጃን ማሰባሰብ በስራ ባልደረቦቼና በሀለቆቼ ይበረታታል	1	2	3	4	5	6	7
B 7	ሰራተኞች ስዉድድርሲሉ ማስረጃን ይነካካሉ/ይቀያይራሉ	1	2	3	4	5	6	7
B 8	የማስረጃ ጥራትን በመጠበቅ ረገድ ስንፍና አለብኝ	1	2	3	4	5	6	7
A c 1	ሰራተኞች የተቀመጠዉን ግብ ሳይደርሱ ሲቀሩ እራሳቸዉን ይወቅሳሉ	1	2	3	4	5	6	7
A c 2	ሰራተኞች ስዝቅተኛ አፈጻጸማቸዉ ይጠየቁበታል	1	2	3	4	5	6	7

R 1	ሰራተኞች የስራ ጊዜን ይጠብቃሉ	1	2	3	4	5	6	7
R 2	ሰራተኞች የህብረተሰቡን ጤና ደረጃ ለማሻሻል ሀላፊነት ይሰማቸዋል	1	2	3	4	5	6	7
R 3	ሰረተኞች ስራዎቻቸዉን ይመዘግባሉ መዝገቦችንም ይጠብቃሉ	1	2	3	4	5	6	7

K1. ከዚህ በታች ባሉት ላይ በየወሩ ማስረጃ የሚሰበሰብበትን ምክንያቶች ብያንስ ሶስት ግለጽ

K1A. በሽታ

1.

2.

3.

K1B. ክትባት

1.

2.

3.

K2. የህዝብ ብዛት ማስረጃ ለምን አስፈለገ

1.

2.

3.

CS. የማስረጃ ጥራትን የምንለካበት መንገዶች ሶስት ይጥቀሱ!

1.

2.

3.

የማስረጃ ሰብሳቢ ስም-------------------------------ፊርማ----------------

የማስረጃ ሰብሳቢ ሙያ---------------------------

የማስረጃ ሰብሳቢ አድራሻ----------------------

ማስረጃ ማሰባሰብ ያስቀበት ሰአት-------------------

ማስረጃ ማሰባሰብ የወሰደዉ ጊዜ ድምር-----------

እናመሰግናስን!

3. Afaan Oromo version data collection questionnaires
Seensa

Qorannoon of danda'e kun, kan sektara fayyaa keessatti sirna bulchiinsa odeeffannoo fooyyessuudha. Kaayyoon qorannoo kanaa tarkaanfiilee sirna odeeffannoo fi qulqullina ragaa fooyyessuuf oolan akka qophaa'an gargaaruudha. Adaraa yaada keessan amanamummaadhaan ibsaa. Odeeffannoon isin nuuf kennitan iccitiin isaa kan eegamuu fi bifa gabateen dhiyaachuudhaan alatti nama kamiifuu kan hin qoodamneedha. Deggarsaa fi gamtoomina isin qo'annoo kana guutuuf gootan ni dinqisiifanna.
Galatooma!

Kutaa I. gaaffilee amaloota hawaasummaa waliin wal qabatan

ID1.	Maqaa dhaabbata fayyaa__________________ID2. maqaa depaartimentii____________		
ID3.	Aanaa_____________		
ID4.	Gosa dhaabbatichaa:	1. Kellaa fayyaa 2. Buufata fayyaa 3. Hospitaala	
DD1.	Waamama nama waraqaa gaaffii kana guutuu (**deebiikee itti mari**)	1. Qindeessaa garee yaala deddeebii 2. Qindeessaa phaarmaasii<br>3. Bulchaa dhaabbata fayyaa 4. Hoogganaa waardii 5. Qondaala HMIS<br>6. Gaggeessaa garee laaboraatoorii 7. kan biro yoo jiraate (adda baasi__________________)	
DD2.	umrii_________________		
DD3	Bara tajaajilaa_____________		
DD4.	Miindaa _____________________birrii Itiyoophiyaatiin		
DD5.	saala:	1. Dhiira 2. Dhalaa	
DD6.	Iddoo jireenyaa:	0. Baadiyyaa 1. Magaalaa	
DD7.	Sadarkaa	1. Dipilooma 2. Digrii jalqabaa 3. Digirii lammaffaa 4. Digrii sadaffaa	

	barnoota:	5. kan biro__________	
DD8.	Gosa ogummaa:	1. Hojjettuu existeenshinii fayyaa 2. Nursii deessiftuu 3. Nursii wal'aansaa 4. Ogeessa eegumsa fayyaa naannoo 5. Ogeessa qorichaa 6. Ogeessa laaboraatoorii 7. Qondaala fayyaa 8. Doktora 9. Nageenyummaa iddoo hojii 10. Sirna nyaataa 11. Saayikaatirii 12. Infoormaatiksii fayyaa 13. Ogeessa raadiyoolojii 14. kan biro yoo jiraate_____________	

Amma nuti hangam akka isin cimsitanii hojiilee tokko tokko kanneen isiniifi dhaabbata keessaniin hojjetaman irratti walii galtan yaada keessan baruu barbaanna. Yaada keessan iskeelii yaadaa irratti ibsuu qofa malee deebiin sirrii yookiin dogoggora jettan hin jiru. Iskeelichi hangam akka isin cimsitanii yaadichatti amantan kan sakatta'uu fi reenjiin isaas cimsee itti walii hin galu(1) -cimseen itti walii gala(7) tti deema. Jalqaba yaada sanatti waliif galuu fi waliigaluu dhabuu keessan murteessuu qabdu. Lammaffaarratti cimina itti waliif galuu fi walii galuu dhabuu adda baastu. Yoo himichatti walii hin galtan ta'e, iskeelii gara harka bitaa jiru fayyadamuudhaan hagam tokko akka itti walii hin galin jechuunis cimsee itti walii hin galu (1), hanga tokko itti walii hin galu (2) fi itti walii hin galu(3) kan jedhu murteessuudhaan deebii keessan itti maraa. yoo itti walii galuu fi walii galuu dhabuu keessan murteessuun isin rakkise 4 tti maraa. Yoo himichatti kan waliif galtan taate, iskeelii gara mirgaa jiru fayyadamuudhaan hagam tokko akka itti waliif galtan jechuunis:-waliin gala (5), hanga tokko walii gala (6) ykn cimseen itti walii gala(7) jechuun erga murteessitanii booda deebii keessanitti maraa. Adaraa hubadhaa himoota hundumaa wajjin walii galuu fi walii galuu dhabuu dandeessu; haaluma walfakkaatuun ciminni walii galuu fi itti walii galuu dhabuu keessaniis garaa garaa ta'uu mala. kanaafuu yeroo walii galuu fi walii galuu dhabuu ibsitan garaagarummaan yaadaa ni eegama. Akka isin garaagarummaa amantaa qabdan sana nuuf ibsitan isin jajjabeessina. Odeeffannoon kun iccitiin isaa kan eegamuu fi bifa gabaasaatiin qindaa'ee dhiyaachuun alatti nama tokkoofiyyuu kan hinqoodamnedha. Adaraa deebii keessan amanamummaadhaan filadhaa. Iskeelii 1-7 jiru keessaa hangam akka walii galtan filadhaa.

Kutaa II. Sakatta'iinsa sababoota teekinikaa itti fayyadama odeeffannoo fayyaa idilee waliin walqabatan

1. Cimsee itti walii hin galu, 2. Hanga tokko itti walii hin galu 3. Itti walii hin galu 4. Itti walii galuu hindidu ittis walii hin galu 5. Waliin gala 6. Hanga tokko waliin gala 7. Cimseen itti walii gala

T.L	Gaaffilee							
1	Tuutni agarsiiftuu sadarkaa eeggate ni jira.	1	2	3	4	5	6	7
2	Foormiin gabaasni itti gabaafamu kan itti fayyadamuuf mijataa ta'ee ni jira.	1	2	3	4	5	6	7
3	Staafiin leenjii qabu kan foormii guutuu danda'u ni jira.	1	2	3	4	5	6	7

Kutaa III. Tuulii sababootni dhaabbataa fi amaloota dhuunfaa waliin walqabatan itti sakatta'aman

1. Departmentii kana keessa ji'oota ja'an 6'n darban keessa leenjii HMIS namni fudhate jira? 0. Lakkii 1. Eeyyee								
2. Istaafiin kompuutera argachuu ni danda'u 0. lakkii 1. Eeyyee, ilaalameera								
3. Istaafiin interneetii argachuu ni danda'u. 0. lakkii 1. Eeyyee								
1. Cimsee itti walii hin galu, 2. Hanga tokko itti walii hin galu 3. Itti walii hin galu 4. Itti walii galuu hindidu ittis walii hin galu 5. Waliin gala 6. Hanga tokko waliin gala 7. Cimseen itti walii gala								
T.Lak	Gaaffilee							
S1.	Qaamni olaanu, duubdeebii idilee karaa gabaasa idilee ragaa irratti hundaa'uudhaan hojjettoota isaaniitiif ni kennu	1	2	3	4	5	6	7
S2.	Qaamni olaanu, qulqullina ragaa gabaasa ji'aa keessa jiruurratti ni xiyyeeffatu.	1	2	3	4	5	6	7
S3.	Qaamni olaanu, superviijinii deggersaa qulqullina ragaa irratti xiyyeeffachuudhaan hojjettoota isaaniif ni taasisu.	1	2	3	4	5	6	7
S4.	Qaamni olaanu, sadarkaa dhaabbata fayyaa fi sadarkaa olaanutti ragaan qulqulluu ta'uusaa yeroo yeroodhaan ni ilaalu	1	2	3	4	5	6	7
S5.	Qaaamni olaanoon, yeroo yeroo dhaan gabaasa qulqullina ragaa ni gabaasu	1	2	3	4	5	6	7
P3.	Istaafiin hojii gaarii hojjetaniif ni badhaafamu	1	2	3	4	5	6	7
A1.	Istaafiin ragaa sassaabuu akka hojii bu'aa hin qabneetti ilaalu	1	2	3	4	5	6	7
A2.	Istaafiin ragaa sassaabuu akka yeroo nama tajaajila kennuu balleessuutti ilaalu	1	2	3	4	5	6	7
E1	Istaafiin hojiilee dhaabbaticha keessatti raawwataman hunda irratti dammaqinaan ni hirmaatu	1	2	3	4	5	6	7

PR	Dhaabbanni fayyaa gamaaggama raawwii hojii qaamota dhimmi ilaalu waliin ni taasisa.	1	2	3	4	5	6	7
U1	Istaafiin bulchiinsa guyyaa guyyaa dhaabbatichaaf ragaa HMIS ni fayyadamu.	1	2	3	4	5	6	7
U2.	Istaafiin manii kaayyatan hordofuudhaaf ragaa giraafii fi gabateedhaan ni mul'isu:	1	2	3	4	5	6	7
U3.	Istaafiin weerara dhukkubaa adda baasuu fi yaaluudhaaf ragaa HMIS tti ni fayyadamu:	1	2	3	4	5	6	7
U4.	Istaafiin dhiyeessaa fi bulchiinsa qorichaatiif ragaa HMIS tti ni fayyadamu	1	2	3	4	5	6	7
U5.	Istaafiin hawaasa barsiisuu fi kakaasuudhaaf ragaa HMIS tti ni fayyadamu	1	2	3	4	5	6	7
B1	Odeeffannoo murtoodhaaf itti hin fayyadamne sassaabuun na jibbisiisa	1	2	3	4	5	6	7
B2	Odeeffannoo sassaabuun na hifachiisa	1	2	3	4	5	6	7
B3	Odeeffannoo sassaabuun anaaf hiikkaa qaba	1	2	3	4	5	6	7
B4	Odeeffannoo sassaabuun miira ragaan raawwii dhaabbatichaa hordofuudhaaf barbaadamuu naaf kenna.	1	2	3	4	5	6	7
B5	Odeeffannoo sassaabuun akka waan narratti fe'ame tokkootti natti dhagayama	1	2	3	4	5	6	7
B6	Odeeffannoo sassaabuun namoota waliin hojjedhuu fi namoota naa ol jiran biratti fudhatama qaba.	1	2	3	4	5	6	7
B7.	Hojjettoonni waldorgomuuf jecha ragaa ni jijijjiiru/itti bubu'u.	1	2	3	4	5	6	7
B8.	Qulqullina ragaa eeguurratti danta-dhabummaan qaba.	1	2	3	4	5	6	7
Ac1	Hojjettoonni galma kaa'ame yeroo hanqatan mataa isaanii of ceepha'u	1	2	3	4	5	6	7
Ac2	Hojjettoonni raawwii gad-aanaa galmeessaniif ni gaafatamu	1	2	3	4	5	6	7
R1	Hojjettoonni yeroo ni kabaju	1	2	3	4	5	6	7
R2	Hojjettoonni sadarkaa fayyaa uummataa fooyyessuuf jecha itti gaafatamummaan itti dhaga'ama	1	2	3	4	5	6	7
R3	Istaafiin waan hojjetan galmeessuudhaan ragaas ni qabatu	1	2	3	4	5	6	7

K1. Kanneen armaan gadiitiif sababoota ragaan ji'a ji'aan itti funaanamuuf yoo xiqqaate sadii ibsaa:

K1A. Dhukkuba

1.

2.

3.

K1B. Talaallii

1.

2.

3.

K2. Ragaan baayyina uummataa maaliif barbaachise?

1.

2.

3.

CS. Karaalee ittiin qulqullina ragaa adda baasan yoo xiqqaate sadii (3) ibsaa.

1.

2.

3.

Maqaa guutuu Nama ragaa funaanuu: ______________________________Mallattoo:________

Ogummaa nama ragaa guuruu_____________________

Bilbila nama ragaa guuruu__________________

Sa'aa raga guuruun itti xummurame: ____________

Waliigala sa'aa raga guuruuf fudhate_____________

Galatooma!

4. Check-List for Departments

**Part II. Data quality assessment form at the health facility level** **Part II.1. Data recording**		
Q1	Does this department keep copies of the Routine HMIS reports, which are sent to the higher level?	1. Yes 0. No, if no, go to Q3
Q2	Count the number of Routine HMIS reports that have been kept at the department for the last twelve months____________	
Q3	Does this department keep registration book of different activities?	1. Yes 0. No, if no, go to Q5

Part II.2: Data Accuracy Check

Q4. If yes to Q3, take the three indicators/data items randomly and find the information in the registration book of department for the selected two months. If the department does not keep copies of the monthly report, obtain copies at the next higher level and complete the following.

		Month: December/2019		Month: January 2020		
	Data Items	#from Registration book	#fromReport	#from Registration book	# fromReport	
1						
2						
3						
	Total score=					

Part II.3: Data Completeness

Part II.3a: Source document completeness

Take the last 15 entries recorded in the source document for each reporting period and check if all the data elements relevant to all the selected indicators are filled in. If the total entries are less than 15, use the available entries. a. Complete means that the source document contains all the data elements relevant to the selected indicator, b. incomplete means that there is missing data elements relevant to the selected indicators.

		**January**				**February**				**%total**
	**indicators**	**a**	**B**	**C**	**d**	**a**	**b**	**C**	**d**	**e=d+d/2**
**1**										
**2**										
**3**										
	**Total=**									

Part II.3b: Report completeness

Q5	How many relevant data items does the department need to report on the Routine HMIS report for the selected indicators? This number does	

	not include data items for services not provided by this health department/unit.	
Q6	Count the number of data items that are supposed to be filled in by this department but left blank without indicating “0” in the selected month’s report.	
**Part II.4. Timeliness**		
Q7	What is the deadline for submitting monthly report for this health department?	
Q8	Does this department/unit submit a report on time?	1. Yes 0. No
Q9	If yes to Q8, check the date of submission for two months monthly reports to answer Q9 (December 2012and January2020).	

	Months	within the reporting period interval	out of the reporting period interval
Q9	December 2019	1. Yes, observed 0. No	1. Yes 0. No
	January 2020	1. Yes, observed 0. No	1. Yes 0. No
